# Factors influencing implementation of a care coordination intervention for cancer survivors with multiple comorbidities in a safety-net system: an application of the Implementation Research Logic Model

**DOI:** 10.1186/s13012-023-01326-8

**Published:** 2023-12-04

**Authors:** Serena A. Rodriguez, Simon Craddock Lee, Robin T. Higashi, Patricia M. Chen, Rebecca L. Eary, Navid Sadeghi, Noel Santini, Bijal A. Balasubramanian

**Affiliations:** 1 Department of Health Promotion & Behavioral Sciences, School of Public Health, The University of Texas Health Science Center at Houston (UTHealth Houston), 2777 North Stemmons Freeway, Dallas, TX 75207 USA; 2 Center for Health Promotion & Prevention Research, UTHealth Houston School of Public Health, 7000 Fannin, Houston, TX 77030 USA; 3 UTHealth Houston Institute for Implementation Science, 2777 North Stemmons Freeway, Dallas, TX 75207 USA; 4 Department of Population Health, University of Kansas Medical Center, 3901 Rainbow Blvd, Kansas City, KS 66160 USA; 5 University of Kansas Cancer Center, 2650 Shawnee Mission Parkway, Westwood, KS 66205 USA; 6 Peter O’Donnell Jr. School of Public Health, University of Texas Southwestern Medical Center, 5323 Harry Hines Blvd, Dallas, TX 75390 USA; 7 Department of Family and Community Medicine, University of Texas Southwestern Medical Center, 5939 Harry Hines Blvd., Suite 303, Dallas, TX 75390 USA; 8 Department of Internal Medicine, University of Texas Southwestern Medical Center, 5323 Harry Hines Blvd, Dallas, TX 75390 USA; 9 Harold C. Simmons Comprehensive Cancer Center, University of Texas Southwestern Medical Center, 6202 Harry Hines Blvd, Dallas, TX 75235 USA; 10 Parkland Health, 5200 Harry Hines Blvd, Dallas, TX 75235 USA; 11 Department of Epidemiology, Human Genetics & Environmental Sciences, UTHealth Houston School of Public Health, 2777 North Stemmons Freeway, Dallas, TX 75207 USA

**Keywords:** Implementation outcomes, Cancer survivorship, Safety-net healthcare system, Implementation, Qualitative study, Serena a. Rodriguez and Simon Craddock Lee are joint first authors

## Abstract

**Background:**

Under- and uninsured cancer survivors have significant medical, social, and economic complexity. For these survivors, effective care coordination between oncology and primary care teams is critical for high-quality, comprehensive care. While evidence-based interventions exist to improve coordination between healthcare teams, testing implementation of these interventions for cancer survivors seen in real-world safety-net settings has been limited. This study aimed to (1) identify factors influencing implementation of a multicomponent care coordination intervention (nurse coordinator plus patient registry) focused on cancer survivors with multiple comorbidities in an integrated safety-net system and (2) identify mechanisms through which the factors impacted implementation outcomes.

**Methods:**

We conducted semi-structured interviews (patients, providers, and system leaders), structured observations of primary care and oncology operations, and document analysis during intervention implementation between 2016 and 2020. The practice change model (PCM) guided data collection to identify barriers and facilitators of implementation; the PCM, Consolidated Framework for Implementation Research, and Implementation Research Logic Model guided four immersion/crystallization data analysis and synthesis cycles to identify mechanisms and assess outcomes. Implementation outcomes included appropriateness, acceptability, adoption, and penetration.

**Results:**

The intervention was appropriate and acceptable to primary care and oncology teams based on reported patient needs and resources and the strength of the evidence supporting intervention components. Active and sustained partnership with system leaders facilitated these outcomes. There was limited adoption and penetration early in implementation because the study was narrowly focused on just breast and colorectal cancer patients. This created barriers to real-world practice where patients with all cancer types receive care. Over time, flexibility intentionally designed into intervention implementation facilitated adoption and penetration. Regular feedback from system partners and rapid cycles of implementation and evaluation led to real-time adaptations increasing adoption and penetration.

**Discussion:**

Evidence-based interventions to coordinate care for underserved cancer survivors across oncology and primary care teams can be implemented successfully when system leaders are actively engaged and with flexibility in implementation embedded intentionally to continuously facilitate adoption and penetration across the health system.

**Supplementary Information:**

The online version contains supplementary material available at 10.1186/s13012-023-01326-8.

Contributions to the literature

- This study uses the IRLM to demonstrate the evolving relations between determinants, implementation strategies, mechanisms, and implementation outcomes and shows that determinants are dynamic and change over time to influence multiple implementation outcomes.
- We argue that flexibility of implementation is necessary to accelerate translation of EBIs in real-world settings and does not necessarily constitute a decrease in fidelity to the EBI.
- We demonstrate that implementing a system-level EBI to coordinate care between oncology and primary care for cancer survivors with chronic conditions in a safety-net healthcare system is appropriate and acceptable to patients and stakeholders.

## Introduction

Early detection and treatment advances are driving steady increases in the number of cancer survivors. In many cases, living with cancer has become similar to living with common chronic conditions such as diabetes and heart disease. Most patients with cancer also have three or more chronic conditions [1, 2] requiring coordinated care between oncology, primary care, and other specialties. Primary care can play an important role in providing comprehensive, coordinated care for all conditions, including cancer. In fact, national cancer survivorship guidelines recommend that patients with cancer, known as cancer survivors, need primary care clinicians (PCC) as part of their care team because PCCs play an important role in providing comprehensive, whole person care [3–5]. To achieve these goals, communication and coordination between primary care and oncology are paramount [6–9]. Although central to the Institute of Medicine’s recommendations made nearly 20 years ago [10], the field is unclear how to ensure cancer survivors stay connected with primary care from start of cancer treatment and throughout their cancer survivorship journey.

Well-established evidence from primary care settings demonstrates the effectiveness of using patient registries and designated care coordinators for improving patient outcomes for many chronic conditions such as diabetes and hypertension [2, 11–13]. Patient registries enable identification of all patients with specific conditions (e.g., breast cancer, diabetes) to proactively plan care delivery for timely provision of preventive and chronic disease care. Care coordinators play a critical role in managing referrals and connections between specialties and in ensuring that relevant clinical information to manage patients’ care is available and accessible to clinicians at point of care. However, few trials have studied implementation of these evidence-based interventions among patients living with cancer and chronic conditions, particularly in safety-net healthcare settings.

Project CONNECT implemented these effective care coordination interventions (care coordinator plus patient registry) among cancer survivors with chronic conditions in an urban, integrated safety-net health system that serves disproportionately under- and uninsured ethnic/racial minority [14]. The aims of this study are to (1) identify factors influencing implementation of Project CONNECT and (2) identify mechanisms through which the factors influenced implementation outcomes.

## Methods

This multi-method qualitative study was embedded within a pragmatic trial that tested the implementation of a multicomponent evidence-based intervention aimed at enhancing care coordination for breast and colorectal cancer survivors with chronic conditions [15]. Study procedures were approved by the University of Texas Southwestern Institutional Review Board (STU 102015–090), the University of Texas Health Science Center at Houston, and by Parkland Health Office of Research Administration, and reporting follows the Standards for Reporting Qualitative Research guidelines [16].

### Setting

This study was conducted at Parkland Health (Parkland), the safety-net health system serving Dallas County, TX, USA [17, 18]. “Safety-net” healthcare systems are those that deliver healthcare primarily to uninsured, Medicaid, and other low-income and vulnerable patient populations [19]. Parkland includes a network of 13 primary care clinics located in predominantly under resourced, ethnic/racial minority communities across Dallas County, and a centrally located main campus. The main campus consists of an inpatient hospital, outpatient surgery center, and specialty care clinics, which include multidisciplinary cancer clinics (i.e., medical, surgical, and radiation oncology clinics).

Breast and colorectal cancer are the top two types of cancers treated at Parkland. Twenty-four percent of patients with breast cancer present with stages 3 or 4 breast cancer compared to 10% nationally; 61% of patients with colorectal cancer present with stages 3 and 4 cancer compared to 45% nationally [20].

### Evidence-based intervention components and implementation strategies

Project CONNECT was a multicomponent evidence-based intervention and included (1) an electronic medical record (EMR)-based patient registry and (2) a care coordinator [15]. The registry identified patients diagnosed with stages I–III breast or colorectal cancers plus one or more of the following chronic conditions: diabetes, hypertension, heart disease, chronic kidney disease, and/or chronic lung disease. The care coordinator was a registered nurse employed by Parkland who helped connect study eligible cancer survivors to primary care by facilitating appointments with primary care and coordinated care for patients between oncology and primary care. Strategies identified a priori to implement the intervention components into clinical practice including the following: identifying champions, changing records systems, creating new clinical workflows, and flexibility in implementation (Table 1) [15].

**Table 1:** Intervention components, functions, and implementations strategies

**Intervention component** Functions	**Implementation strategies** Operationalization in Project CONNECT
**Patient registry** Capture patients with the following attributes: • Stages I–III breast or colorectal cancers • Diagnosed with ≥ 1 of the following chronic conditions: diabetes, hypertension, chronic lung disease, chronic kidney disease, and/or chronic heart disease • Presenting at medical oncology clinic **Full-time nurse coordinator with competencies in care coordination co-located in oncology and primary care** Coordinate care for patients identified through registry: • Establish relationships with providers and staff in oncology and primary care • Facilitate appointment scheduling between primary care and oncology • Coordinate lab tests and appointment referrals to specialty care • Track appointments and results • Assign PCP to patients with cancer presenting through the emergency department Ensure continuity of care between treatment and survivorship phases • Notify primary care providers (PCPs) of patient completion of cancer treatment and transition to survivorship phase • Initiate treatment summary and follow-up guidelines and synthesize patients’ medical and cancer history	**Identify champions**^a^ Identify providers, staff, and other system stakeholders to advocate for interventions, support intervention implementation, and provide ongoing feedback **Change record systems**^a^ Incorporate patient registry into existing electronic health records system using the EPIC Reporting Workbench **Create new clinical workflows**^a^ Develop new clinical pathways between primary care and oncology (or emergency department, primary care, and oncology) incorporating the nurse coordinator to improve care coordination and clinical outcomes for patients **Flexibility in implementation** Respond in real time to implementation challenges and/or opportunities based on stakeholder feedback

^a^Adapted from Powell et al. (2015) [21]

### Guiding theoretical and conceptual frameworks

The practice change model (PCM) and the Consolidated Framework for Implementation Research (CFIR) are determinant frameworks that guided data collection to identify barriers and facilitators of implementation [22, 23]. The PCM includes four elements (e.g., internal motivators, external motivators, resources, and opportunities for change) and depicts how these multi-level elements can impact intervention implementation in healthcare settings over time [22]. The CFIR is a menu of individual-, program-, and organizational-level constructs consolidated from 19 theories and models related to intervention implementation [24]. The constructs are organized into five overarching domains: intervention characteristics, outer setting, inner setting, characteristics of individuals, and process. These frameworks are highly complementary. PCM grounded our focus on drivers of practice operations and potential interactions, while CFIR helped us attend to relationships [25] between our intervention, actors in the practice, and the structure and sequence of care delivery to maximize learning from our real-world setting [26].

Proctor and colleagues’ taxonomy of implementation outcomes [27] and the Implementation Research Logic Model (IRLM) [28] informed our data analysis and synthesis [27].This study used qualitative data to assess two implementation outcomes at the patient and provider levels (i.e., intervention acceptability and appropriateness) and two outcomes at the organizational level (e.g., intervention adoption and penetration). Acceptability is defined as patient and/or provider satisfaction with the intervention, and appropriateness is defined as the perceived fit of the intervention in the setting [27]. Adoption is defined as the initial uptake of the intervention; penetration is defined as the integration of an intervention within a clinical team, which is similar to the concept of “reach” in Glasgow’s RE-AIM framework [29]. Adoption and penetration were assessed longitudinally during intervention implementation (Phase 2, see below) allowing assessment of continued adoption or utilization of the intervention beyond initial uptake. Finally, the IRLM is a visualization tool to depict causal pathways between intervention components, determinants (i.e., barriers and facilitators) of implementation, implementation strategies, mechanisms of action, and implementation [28]. Mechanisms of action define *how* implementation strategies operate to influence outcomes. We used the IRLM to elucidate the relationships between determinants, mechanisms, and implementation outcomes.

### Data collection

Trained investigators (R. T. H., P. M. C., S. C. L.) collected qualitative data throughout intervention implementation from September 2016 through June 2020. This included two phases of the study. Phase 1 of data collection occurred from September 2016 to September 2018, pre-intervention implementation. Phase 2 occurred from September 2018 to June 2020 during intervention implementation.

We used purposive sampling to select clinical team members who varied by their roles and specialty to identify barriers and facilitators to delivering coordinated care for patients with cancer and chronic conditions. Study participants included clinicians (e.g., physicians, nurse practitioners), clinic staff (e.g., nurses, care coordinators, social workers, financial services coordinators), and health system leaders (e.g., unit managers, clinic managers, and medical service chiefs) in oncology, primary care, and specialty care. We recruited multiple participants for each role and unit to solicit diverse perspectives.

Data sources included the following: (1) documents, (2) structured observations and field notes, and (3) semi-structured interviews with patients, providers, staff, and leaders from multiple departments across the integrated safety-net system.

#### Documents

Documents included meeting notes, policies and procedures, correspondence among stakeholders, EMR screenshots, patient-facing materials, tools and checklists, and other resources. Documents were requested from providers, staff, and leaders, and they were also offered unsolicited by interviewees and observed stakeholders to clarify processes, provide supplementary information, or serve as historical records.

#### Structured observations and field notes

We used structured observation guides to facilitate consistent data capture of care coordination and practice change processes [30]. Exemplar domains and questions included the following: evidence of team-based care (e.g., do oncology providers discuss other conditions or comorbidities?), documentation of practice (e.g., where do oncology providers document information related to the survivorship care plan or referrals for follow-up care after discharge?), patient access to information (e.g., do providers tell patients what to do in the event of acute needs?), continuity of care (e.g., how are subsequent appointments scheduled?), and team-based care (e.g., to what extent do providers engage patients in taking an active role in their care?). We also selected sites for observation to capture the patient pathway and provider/staff movement through the care coordination process (e.g., registration and intake areas, patient-provider interactions, provider-staff interactions and work areas, and nurse navigation, referral, and case management processes) [31].

#### Semi-structured interviews

Interviews were semi-structured to guide the interviewer through pre-planned topics while allowing for follow-up questions tailored to participant feedback and for additional unplanned questions to be incorporated as appropriate. Interview guides were iteratively developed by investigators and adapted to role and clinical unit. We anticipated barriers and facilitators to implementation based on the CFIR [23] and PCM [22] and, accordingly, focused the interviews on domains including the following: care coordination processes between oncology and primary care, perceptions of the role of the nurse coordinator and registry (interventions), challenges or gaps in care for cancer survivors with chronic conditions, communication about policies and procedures within clinics, EMR documentation and challenges, delineation of roles and expectations between oncology and primary care providers and staff, and patient feedback about areas of confusion or concern. Prior to participating in an interview, informed verbal consent was obtained from all study participants. Patients received a US $25 gift card in appreciation for their time. In accordance with Parkland policy, employees were not provided with an incentive to participate in research.

### Data analysis

#### Immersion-crystallization processes

Data analysis proceeded in four immersion-crystallization cycles, or repeated exposure to and synthesizing of data, to identify themes and categories (Supplementary Materials) [32].

In cycle 1, the team developed two deductively driven thematic codebooks based on interview guide topics, pre- and post-intervention phases, and a preliminary review of documents (*n* = 259 unique documents), field observations (*n* = 11), and interview transcripts (*n* = 140). Additional emergent themes were incorporated into the initial codebook drafts for the first 10% of transcripts, and the finalized codebook was used for remaining transcripts. Codebooks for the two phases included many of the same codes; however, each also included additional unique codes given differences in thematic foci and emergent findings during each phase. For example, Phase 1 codes included existing barriers to care coordination, organizational structure, and processes; Phase 2 codes included patient experiences, acuity of care, and transitions in care. All coding was completed in NVivo 12.0 (QSR, Australia). After coding all data, the team created node reports, summaries of data collected, and exemplar quotes for each code and identified codes tying together steps in the cancer care continuum to the intervention components: care coordination, survivorship planning, intervention, and intervention impact.

In analysis cycle 2, the team applied codes from PCM and CFIR to the selected node reports focusing on identifying organizational inner setting characteristics, system resources, stakeholder motivations, and opportunities for change. The purpose of this cycle was to understand how and why care coordination processes occurred pre- and post-intervention. In cycle 3, the team returned to the findings from cycles 1 and 2, coding for in order to describe how the intervention components and care coordination processes mapped to implementation outcomes. Implementation outcomes were assessed from qualitative data; most validated quantitative implementation outcome measures were not available at the start of this study. In cycle 4, the team met weekly to interpret findings and synthesize data linking implementation strategies, determinants, mechanisms, and outcomes using the IRLM.

## Results

**Fig. 1:**
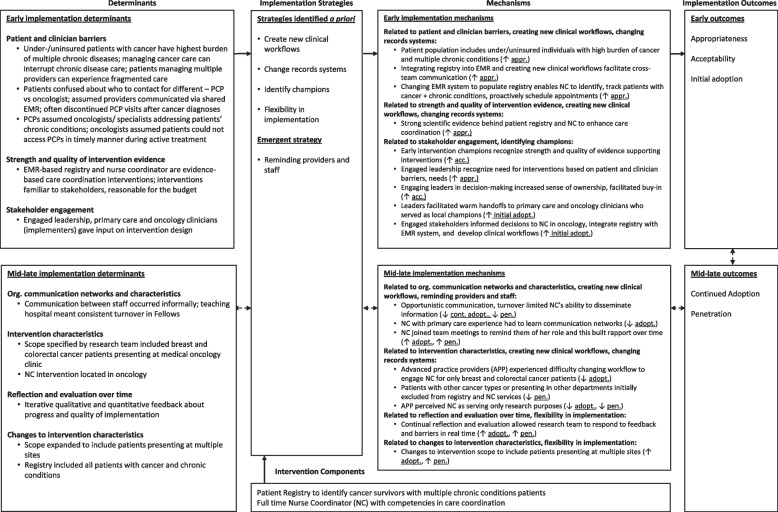
Determinants, implementation strategies, and mechanisms linked with implementation outcomes

Figure 1 depicts the determinants, implementation strategies addressing the determinants, and observed mechanisms influencing each implementation outcome, and Table 2 provides illustrative quotes for determinants.

**Table 2:** Determinants and exemplar quotes

Determinant	Exemplar quote
Patient and clinician barriers	“I thought maybe they already knew the information on the paperwork because they’re supposed to connect through the internet which is like a circle. You know, your primary, your cancer, your research doctor, your breast cancer—all these people should be communicating” — *Patient* “They are getting refills from Oncology for diabetes or other, you know, chronic medical problems. Then they don’t feel the need to come to a PCP because they are getting medication refills done by Oncology. Then they will not feel the need to come over here” — *Primary care provider*
Stakeholder engagement	“I think that some of these decisions are not local decisions – they are enterprise decisions. For example, it goes back to what panel of patients is appropriate for primary care physicians in order to allow them to better communicate, and what is the role of navigators in those settings? Because not necessarily everything has to be communicated through a physician to physician; some communications could be facilitated by a navigator. I think one part of it is that utilizing navigators is better for us, because our navigators can communicate effectively potentially with the primary care physician…So as we develop our navigation program, this can improve the communication between the clinics as well” — *Administrator*
Organizational communication networks and characteristics	“Even if there’s no new information, [the nurse coordinator] could…join our meeting, have one slide, talk for two to five minutes and say ‘hey guys…this is who I am.’ Employees do better when they know the person, like seeing [the nurse coordinator], what does she look like, hearing her, making connections, improving engagement. So, then they are like, ‘oh yeah, this is what I do when my patient doesn’t have a PCP’” — *Oncology provider*
Intervention characteristics	“The most important thing about [the nurse coordinator]…it was not just her knowledge, it was the fact that she is just a unique person. She was a go-getter. She always thought about ‘how can we do things better?’ She was very thorough” — *Administrator*
Reflecting and evaluating over time	“I am sending her [a direct message via the EHR] and she is responding and saying, ‘yes, we can get it.’ It’s a huge help. So now our environment has changed from feeling completely unsupported to now actually having this backdoor to PCPs, which is a huge help” — *Oncology provider*

### Appropriateness and acceptability

The Project CONNECT intervention was appropriate for the safety-net healthcare setting and acceptable to system leaders. Three determinants (patient and clinician barriers, scientific evidence supporting the interventions, and engaged stakeholders) influenced appropriateness and acceptability primarily through three implementation strategies (creating new clinical workflows, changing records systems, identifying champions) during early implementation.

#### Patient and clinician barriers

The intervention addressed patient and clinician barriers to coordinating care between primary care and oncology. Patients with cancer and chronic conditions described confusion about which clinician to contact (i.e., primary care, oncology, or other specialists) for appointments, medication refills, questions, or concerns. Patients also assumed their providers were actively communicating through the shared EMR system about, for example, changes to medications or their treatment progress. Although clinicians documented these issues in the EMR, they were not proactively communicating across teams unless specific actions were warranted.

Clinicians also expressed challenges caring for patients with cancer and chronic conditions. Primary care clinicians noted patients often did not continue seeing their primary care providers after cancer diagnoses. They then assumed oncologists or other specialists were addressing patients’ chronic conditions. Oncology clinicians, on the other hand, felt patients could not access primary care appointments in a timely manner during active treatment, and they would therefore fill prescriptions for chronic conditions, sometimes for up to 1 year. Changing the EMR system and creating new clinical workflows as implementation strategies facilitated development of the patient registry, which enabled the nurse coordinator to identify patients with cancer and chronic conditions, to track them through their course of oncology treatment, and to proactively schedule appointments, thus addressing both patient and provider needs.

#### Strength and quality of intervention evidence

Oncology and primary care system leaders agreed the selected interventions were appropriate for their setting to address these patient needs, and leaders supported implementation based on the strength of the scientific evidence behind registry and nurse coordinator interventions in enhancing care coordination. These early champions recognized the interventions were familiar to clinical team members, had been deployed in other contexts across Parkland Health, and were reasonable to budget with the assistance of research funds. Identifying and engaging the champions further increased intervention acceptability.

#### Stakeholder engagement

The CONNECT intervention was designed with input from Parkland primary care and oncology leaders who were aware of patient and clinician barriers caring for patients with cancer and chronic conditions. Primary care and oncology system leaders informed the decision to have the nurse coordinator located in the oncology clinic rather than in primary care practices, arguing that patients with chronic conditions in “active” cancer treatment needed continuity with primary care for their chronic disease management needs. System leaders emphasized the need for care coordination from the time of cancer diagnosis, not just after end of active treatment. Over the course of the intervention, leaders proactively identified two nurse coordinators to fill the role. Thus, engaging system leaders in designing and implementing key intervention components facilitated a sense of ownership and influenced intervention appropriateness and acceptability.

### Adoption and penetration

All physicians and advanced practice providers of the Parkland oncology clinic adopted the intervention initially. Initial adoption was facilitated by active stakeholder engagement and leadership support. System leaders facilitated warm handoffs (i.e., provider-to-provider exchange) [33] between primary care and oncology clinicians who served as local site champions to facilitate adoption (aka Parkland implementers). Primary care implementers identified gaps in chronic disease monitoring and management for patients in active cancer treatment. They shared clinical expertise to change the EMR system integrating the patient registry functionality into the EPIC Reporting Workbench. Primary care and oncology care teams both actively engaged with the research team in developing new clinical workflows establishing the nurse coordinator’s role in coordinating care. In turn, the research team was able to provide technical assistance related to the evidence base to inform implementer efforts.

As the intervention progressed over time, there were barriers to continued adoption which then also limited penetration. Two determinants (organizational communication networks and characteristics, intervention characteristics) limited continued adoption and penetration of intervention components during the mid-implementation phase of the study. Reflecting on and evaluating the implementation process and the flexibility embedded in implementation allowed the research team to be responsive to stakeholders, to identify new implementation strategies (e.g., remind providers and staff), and to ultimately facilitate continued use of the intervention and penetration over the course of implementation.

#### Organizational communication networks and characteristics

Organizational communication networks and characteristics impacted continued adoption. Communication about day-to-day activities related to the intervention occurred informally during staff huddles and often via word of mouth limiting dissemination of the intervention across the oncology clinic where the nurse coordinator was embedded. For example, the informal nature of team communication about the intervention limited the nurse coordinator’s ability to share information about the different ways she could support care coordination for cancer patients with chronic conditions. In addition, the safety-net system, a teaching hospital, also experienced frequent turnover in oncology fellows and staff. Without consistent communication and interaction with the nurse coordinator, many did not know the intervention was available to their patients. Compounding one another, these determinants led to limited staff and provider knowledge about the interventions and limited continued utilization of the intervention by oncology providers.

#### Intervention characteristics

Characteristics of the nurse coordinator also influenced continued adoption. At study rollout, a primary care registered nurse filled the role. Because the coordinator intervention was located in oncology, it took time for the primary care nurse to learn the communication networks and to integrate within the oncology team. One year into the study, the nurse left Parkland, and system leaders identified a seasoned oncology nurse to assume the nurse coordinator role. Her familiarity with oncology team members and clinic processes increased others’ adoption of the nurse coordinator intervention.

Barriers to continued adoption coupled with intervention scope further limited intervention penetration. Penetration, in this context, refers to the extent of the intervention’s spread or reach across all oncology clinicians. Advanced practice providers (APPs) expressed difficulty in changing their workflow to engage the nurse coordinator only for breast and colorectal cancer patients (as defined by the research study) when they also experienced challenges connecting patients with other cancer types with their primary care doctors. APPs include licensed nonphysician providers such as nurse practitioners, physician’s assistants, and medical assistants. Although the nurse coordinator was employed by Parkland, the APPs viewed her as only available for the Project CONNECT “research” study, thus focusing only on patients with breast and colorectal cancer. This narrowly defined scope limited the integration of nurse coordinator services into usual clinic workflows and therefore limited intervention penetration. In addition, the nurse coordinator found that not all patients with cancer presented through the medical oncology clinic. In particular, some patients with stage 1 colorectal cancer who started in surgical oncology did not receive follow-up through the medical oncology clinic after surgery, so limiting intervention scope to the medical oncology clinic limited penetration.

#### Reflecting and evaluating overtime

Iterative data collection throughout implementation allowed for reflection and evaluation of the implementation process and allowed the research team and Parkland implementers to respond to stakeholder feedback and implementation barriers. Along with flexibility in implementation, this feedback loop enabled the team to address changes in determinants and adapt or identify new implementation strategies needed to increase adoption and penetration. Based on feedback, the nurse coordinator began attending meetings and huddles to inform new colleagues about her role and to continually remind existing staff and providers about her role. In addition, the research team expanded the intervention’s scope to include patients presenting at multiple sites (e.g., surgical oncology, emergency department) to increase penetration. Finally, changes to the workflow to include all patients with cancer and chronic conditions enabled the nurse coordinator to connect any patient needing chronic disease management to a primary care clinician.

### Flexibility in implementation

The implementation strategy “flexibility in implementation” influenced all four implementation outcomes. Specifically, changes in intervention scope to include patients with any type of cancer increased APP acceptance of the nurse coordinator and therefore their adoption of the intervention. The change in scope allowed the nurse coordinator to further integrate with—or penetrate—the oncology team and expand intervention reach. In addition, continual stakeholder engagement and flexibility ensured intervention components remained appropriate and acceptable throughout implementation.

## Discussion

There is significant interest among researchers, clinicians, health system leaders, and policy makers in identifying optimal ways to coordinate care for cancer survivors, especially those who are under- and uninsured and most likely to have poor health outcomes. This study demonstrated that implementing a system-level evidence-based intervention to coordinate care for cancer survivors with chronic conditions between oncology and primary care in a safety-net health system was appropriate and acceptable to patients and health system stakeholders. While clinicians and clinical staff initially adopted the intervention, continued adoption and penetration of the intervention throughout the clinic were challenging even with support from motivated and engaged health system leaders. This is because the intervention, as designed, experienced challenges integrating into real-world practice. Continual evaluation and reflection allowed the research team to be responsive to stakeholder feedback in real time, to identify emerging determinants, and to develop new implementation strategies to increase acceptability, continued adoption, and penetration. Importantly, flexibility in implementation became a key implementation strategy to address barriers to adoption and penetration over time. While our intervention took place in a safety-net healthcare system in the USA, our findings about how to bridge cancer survivors’ care between PCCs and oncology clinicians are applicable more broadly as comprehensive cancer survivorship care approaches are needed globally for different health care system models [34]. Determinants—such as patient and clinician barriers, lack of stakeholder engagement, the strength and quality of scientific evidence supporting care coordination interventions, and intervention characteristics—have been explored in the context of other interventions and other disease/patient targets [35–37], such as for diabetes and hypertension management. However, our study is the first to examine these determinants in the context of implementing an intervention for patients with cancer receiving care in a safety-net setting. This is significant because an increasing number of patients with cancers such as early-stage breast, colon, and rectal cancer are living decades after their initial diagnosis, thanks to significant advances in early detection and treatments. In such cases, cancer becomes another chronic condition that patients and their clinicians must manage including timely surveillance for recurrence and managing risks associated with cancer treatments and its sequelae. Thus, delineating determinants of adoption and implementation of evidence-based care coordination interventions shown to be effective for routine chronic conditions such as those used in Project CONNECT can aid health systems in coordinating care for patients with cancer and chronic conditions. Importantly, our focus on safety-net health systems has the potential to increase health equity by identifying determinants relevant for patients with signficant social and economic challenges and for under-resourced systems. More cancer survivorship care delivery research embedded in safety-net systems and community health centers is needed to improve care delivery outcomes.

This study shows that determinants are dynamic rather than static constructs and change over time to influence multiple implementation outcomes. While researchers have theorized that determinants may change over time, few studies have embedded longitudinal evaluations such as ours that demonstrate how they change and the ways in which they influence implementation outcomes over time. For example, system leaders affirmed intervention appropriateness and contributed to initial acceptability at multiple levels. As implementation proceeded, it became clear that consistent, iterative engagement with site champions in primary and oncology care was necessary also to ensure continued adoption. Our study design enabled us to observe these changes and influences over time, and our iterative immersion-crystalization data analysis strategy enabled us to identify similar recursive relations between determinants, strategies, mechanisms, and outcomes.

Similarly, this study shows how implementation outcomes are interrelated, influenced by determinants and other outcomes, and how it may be unclear at what point one outcome ends and another begins. Although adoption has often been viewed as the intention or initial decision to use an innovation, we consider adoption as both the initial uptake and continued use of the interventions. For example, while stakeholders initially adopted the nurse coordinator intervention, continued adoption and interaction with the nurse coordinator later waned among APPs, who felt the intervention was only relevant for some of their patients. This limited overall penetration into the system. Flexibility in implementation meant that we could rapidly evaluate and adapt in real time to facilitate continued use of the intervention. Recognizing the need for adaptations and responding to dynamic contexts are recommended strategies when designing for dissemination and sustainability [38]. We hypothesize that the phenomena we captured in variable adoption and penetration may be early determinants of maintenance or institutionalization of an intervention into practice. In fact, in our recent meeting with director of Parkland Global Oncology, we learned that Project CONNECT interventions are still being used at Parkland in a modified form.

Describing these interdependent relations is critically important to keep the field moving forward and for research among healthcare systems as they are complex systems adaptive to internal and external factors. Thus, mechanisms of how strategies address determinants to improve implementation and service outcomes are more likely to be inter-dependent rather than linear. Our study’s design and analytic methods helped bring this reality to light.

Our data analysis strategy and use of the IRLM were fundamental to identifying, defining, often disentangling determinants and strategies, understanding their mechanisms, and linking them to implementation outcomes. Smith et al.’s IRLM has been used to guide design and evaluation of implementation studies, describe implementation barriers and facilitators, list hypothesized mechanisms, and engage stakeholders. [28, 39–51] Our analysis advanced the authors’ recommendation to use the tool to elucidate the evolving relations between determinants, implementation strategies, mechanisms, and implementation outcomes [28]. Few studies collect the data needed to describe these changes over time. Our analysis exemplifies why continuous process evaluation data are needed longitudinally and why investing in mixed-method, comprehensive, and longitudinal evaluation data is crucial for rigorous implementation research.

Our study also sheds light on the balance between degree to which an intervention is delivered as intended, i.e., implementation fidelity [52] and flexibility needed for integrating the intervention in real-world settings [53]. We argue that flexibility of implementation is necessary to accelerate translation of evidence-based interventions in real-world settings, and it does not necessarily constitute a decrease in fidelity to the evidence-based intervention. Both fidelity and flexibility are needed and can co-occur in equilibrium such that key functions of evidence-based interventions are implemented with fidelity, but the forms of the interventions themselves may differ across settings, or changes may be made to intervention forms in response to contextual barriers [54]. As shown in our study, implementation flexibility enhanced adoption and penetration of the intervention. This may be a critical nuance that bears further scrutiny and may be a key ingredient in increasing uptake of evidence-based interventions into real-world settings.

### Study limitations

The onset of COVID in North Texas disrupted elective care across the Parkland system, oncology clinic teams pivoted to telephone appointments, and the nurse coordinator was able to continue work remotely. Although direct research observation was temporarily interrupted, our relationships with key stakeholders enabled the research team to continue to collect data through email and telephone exchanges. In addition, a challenge we did not anticipate, but did document, was that annual updates to the Epic EMR could also “break” links even to existing Epic functionality, such as the Reporting Workbench, and needed to be monitored to ensure registry tools remained active.

### Future directions

While our analysis here reports key determinants affecting implementation outcomes, it is yet unclear how increased adoption and penetration may influence team processes supporting care coordination that we did not observe. Although the evidence base for care coordination interventions is strong, the field’s understanding of how factors relevant to local settings shape implementation is still emerging [13]. Forthcoming analyses of Project CONNECT intervention outcomes at the patient and system level could facilitate examination of maintenance and generate key questions to explore about earlier indicators around post-study intervention sustainability once the trial ended.

Having described implementation outcomes here, subsequent analyses of clinical and patient-reported outcomes will help advance our understanding of how these EBIs may help optimize care for these vulnerable patient populations. Similarly, we did not explicitly set out to assess the effectiveness of a bundled implementation strategy, nor to test the separability of our multicomponent intervention. While future work could mount studies to examine these issues, from the perspective of addressing disparities in survivorship care delivery, implementation research should focus on better characterizing the interface between primary care and oncology and identify strategies to better integrate care delivery for cancer survivors such that the care they receive is seamless and addresses survivorship care guidelines holistically [55].

## Conclusion

Effective and accepted interventions such as using population-based registry to track patients with cancer and chronic conditions and assigning a care coordinator to enhance primary care access can be implemented effectively in safety-net health systems. Adoption and penetration across the system can be further enhanced by allowing flexibility in how health systems choose to implement these interventions. Doing so with active and continual engagement of patient and health system partners presents the most promising approach to quickly translate effective interventions into real-world practice to improve care delivery and health outcomes for cancer survivors.

### Supplementary Information

**Additional file 1: Table 1.** Immersion/crystallization cycles of data analysis. **Table 2.** Codes and themes for immersion/crystallization cycles of data analysis.

## Data Availability

The data used and/or analyzed during the current study are available from the corresponding author on reasonable request.
