# Supplementary material for: Factors influencing implementation of a care coordination intervention for cancer survivors with multiple comorbidities in a safety-net system: an application of the Implementation Research Logic Model
Source: Implement Sci. 2023 Dec 4;18:68. doi: 10.1186/s13012-023-01326-8 (PMC10694894; doi:10.1186/s13012-023-01326-8)
Supplement: Supplementary file 1 — Additional file 1: Table 1. Immersion/crystallization cycles of data analysis. Table 2. Codes and themes for immersion/crystallization cycles of data analysis. [file 13012_2023_1326_MOESM1_ESM.docx]

**SUPPLEMENTARY MATERIALS**

| **Immersion/crystallization cycles of data analysis** | | |
| --- | --- | --- |
| **Cycle of Analysis** | **Activity** | **Purpose** |
| 1 | Coding of Phase 1 and 2 data using a deductively-driven codebook of themes (e.g., “survivorship plan”) | Categorize data by relevant themes |
| 2 | Application of CFIR and PCM codes to selected thematic reports (e.g., “care coordination”) | Understand how/why processes occurred |
| 3 | Application of implementation outcomes (e.g., “acceptability”) to cycle one and two findings | Describe how intervention elements and care coordination process pre- and post-launch map to implementation outcomes |
| 4 | Group discussions linking determinants, strategies, mechanisms and outcomes organized in the IRLM | Achieve team consensus in interpretation and synthesis |

| **Codes and themes for immersion/crystallization cycles of data analysis** | | | |
| --- | --- | --- | --- |
| **Cycle 1**  **Thematic Codes**  (Deductive, Inductive) | **Cycle 2**  **CFIR and PCM Codes**  (Interpretative) | **Cycle 3**  **Implementation Outcomes**  (Interpretative) | **Cycle 4**  **Group discussion and synthesis** |
| Acuity  Cancer care  Care coordination  During surveillance  During treatment  Care plan experience  Care plan document  Communication  Costs  COVID  EMR/documentation  Illness experience, perceptions, impact  Intervention  Intervention impact  Medication  Non-Parkland experiences  Other health-related activitites, needs  Primary care  Self-care  Specialty care  Roles, expectations | Stakeholder motivation  Resources  External motivators  Opportunities for change  Inner setting  Outer setting | Acceptability  Appropriateness  Feasibility  Adoption  Fidelity  Implementation cost  Penetration  Maintenance  Sustainability | Emergent implementation strategies Factors (determinants) influencing  implementation  Mechanisms |
